# Adenosine A2A receptor agonist ameliorates EAE and correlates with Th1 cytokine‐induced blood brain barrier dysfunction via suppression of MLCK signaling pathway

**DOI:** 10.1002/iid3.187

**Published:** 2017-10-12

**Authors:** Ying Liu, Marwan Alahiri, Bianca Ulloa, Boxun Xie, Saud A. Sadiq

**Affiliations:** ^1^ Tisch Multiple Sclerosis Research Center of New York 521 W 57th St 4th Fl. New York New York 10019 USA; ^2^ Department of Pathology, School of Basic Medical Sciences Fudan University Yixueyuan Rd. 138 200032 Shanghai China

**Keywords:** Animals, cells, diseases, EAE/MS, endothelial cells, human, monocytes/macrophages

## Abstract

**Introduction:**

Multiple sclerosis (MS) disease activity is associated with blood‐brain barrier (BBB) disruption, which is mediated by inflammatory cytokines released by CD4+ lymphocytes. To assess the effects of adenosine A2A receptors on BBB permeability in vitro and in vivo.

**Methods:**

A2A receptor expression was detected by immunostaining in experimental autoimmune encephalomyelitis (EAE) C57BL/6 mice immunized with myelin oligodendrocyte glycoprotein (MOG)_35–55_, and human MS brain. F‐actin and the tight junction protein Claudin‐5 were assessed in endothelial cells treated with an A2A receptor specific agonist (CGS‐21680) after Th1 cytokine stimulation. EAE mice were divided into control and CGS‐21680 (50 µg/kg, i.p., daily) groups. Disease scores were recorded daily to evaluate neurological impairment. The effects of A2A receptor on inflammation and demyelination were assessed after euthanasia by immunostaining or histology; BBB permeability was measured by sodium fluoride (Na‐F) and FITC‐dextran amounts.

**Results:**

Endothelial A2A receptor was detected in demyelination areas of MS brain samples. In EAE lesions, A2A receptor was expressed in the endothelium in association with immune cell infiltration. Treatment with CGS‐21680 counteracted the effects of Th1 cytokines on endothelial cells in vitro, preventing the reduction of tight junction protein expression and stress fiber formation. The effects of A2A receptor activation were correlated with MLCK phosphorylation signaling repression. In EAE, A2A receptor agonist decreased BBB permeability and inhibited EAE neurologic deficiency in mice.

**Conclusions:**

A2A receptor activation at EAE onset helps reduce the effects of Th1 stimulation on BBB permeability, indicating that A2A receptor mediates BBB function in CNS demyelinated disease.

## Introduction

The blood‐brain barrier (BBB) plays important protective roles in normal neuronal and glial cell functions 1. During neuroinflammation, leukocytes migrate and trigger inflammatory reactions in the neurovascular unit, causing BBB function impairment 2. Therapeutic strategies that restrict leukocyte migration and maintain BBB integrity are critical to neuroprotection.

Multiple sclerosis (MS) is an autoimmune disease of the CNS mediated by T cells. Experimental autoimmune encephalomyelitis (EAE) is a laboratory‐induced disease model used to explore the biology of MS 3. Inflammatory leukocyte infiltration and altered BBB in CNS are the basic pathological hallmarks of both MS and EAE 4. Th1 cytokines are confirmed key factors modulating inflammatory responses and BBB impairment in autoimmune diseases 5. In the neurovascular unit, endothelial tight junctions (TJs) consist of three major classes of transmembrane proteins, namely occludins, claudins, junctional adhesion molecules, and TJs associated proteins, including ZO‐1 4. Claudin‐5 expression is positively correlated with junction tightness in the brain. Altered expression of TJ proteins has been reported in acute and progressive forms of MS 4.

Adenosine binds one or more receptors, acting as a potent endogenous regulator of inflammation 5. Endothelial cells express several or all four known adenosine receptors, including A1, A2a, A2b, and A3 6. Adenosine limits the secretion of the cytokines IL‐6 and IL‐8 by acting on A2A receptors 7. Adenosine also prevents the increase of vascular permeability, and thus promotes endothelial barrier integrity 7. The present study explored the mechanisms by which A2A receptor inhibition affects BBB permeability in the context of the prominent Th1 inflammatory cytokines IL‐1, TNF‐α, and IFN‐γ.

## Material and Methods

### MS brain tissue samples

Paraffin embedded MS brain specimens were kindly provided by the Human Brain and Spinal Fluid Resource Center, VA West Los Angeles Healthcare Center. The MS group consisted of 28 patients with secondary progressive MS and at least 10 years of clinical course. Median age was 60.29 years, ranging between 43 and 79; the male to female ratio was 1:1. The control cohort consisted of 10 patients with bronchiectasis, essential tremor, glaucoma, pneumonia, and Joseph disease. Control brain samples were otherwise normal, except two brains with mild age‐related changes. For controls, median age at death was 70 years, ranging from 50 to 92; the male‐to‐female ratio was 1:1. Average postmortem brain weights of MS and control patients were 1171 ± 36.98 g and 1081 ± 36.27 g, respectively. Average postmortem intervals were 14.71 ± 1.52 h and 9.87 ± 2.63 h for MS and control samples.

### EAE induction

The animal experiments were approved by the St. Luke's Roosevelt Hospital Center IACUC. Chronic EAE was induced in C57BL/6 wild‐type female mice (aged 6–8 weeks) obtained from Jackson Laboratory (Bar Harbor, ME) as follows. The animals were subcutaneously immunized with 200 µg of myelin oligodendrocyte glycoprotein 35–55 (MOG_35–55_, AnaSpec, Fremont, CA, USA) in incomplete Freund adjuvant (IFA, Sigma, St. Louis, MO, USA) plus 8 mg/ml *mycobacterium tuberculosis* (strain H37RA, Difo), with i.v. inoculation of 300 ng *Bordetella pertussis* toxin (PTX, Sigma) at the time of immunization and 48 h thereafter, as previously reported 8. Disability was scored using a 0–13 EAE scale 9, which was modified from the standard 0–5 scale 9. The scorer was blinded to treatment groups. Mice were divided into control (PBS + 10% DMSO, *n* = 15) and CGS‐21680 (50 µg/kg, i.p., daily from the beginning of immunization [Day 0] to Day 20; Abcam, Cambridge, MA, USA; *n* = 15) 9. CGS‐21680 doses were based on previous in vitro studies 10.

### Evaluation of BBB permeability in vivo

Immunized EAE mice (*n* = 5) and CGS‐21680 treated EAE mice (*n* = 5) at 14 days post‐immunization (dpi) were injected intravenously with 50 µl of 2% Na‐F as described previously 1. Briefly, after 30 min, the mice were transcardially perfused with PBS. Brain and spinal cord samples were collected and homogenized in 10 volumes of 50% trichloroacetic acid. After centrifugation, the supernatant was neutralized with 5 M NaOH. Measurements of Na‐F fluorescence were determined at excitation and emission wavelengths of 440 and 525 nm, respectively, on a Spectramax MS microplate reader (Molecular Devices). The data were expressed as amounts of tracer found per mg of tissue.

Immunized EAE mice treated with vehicle (*n* = 5) and CGS‐21680 (*n* = 5) at 14 dpi were used in FITC‐dextran experiments to evaluate BBB permeability in vivo 4. FITC‐labeled dextran (5 mg/ml, 40 kDa, Sigma) was administered by tail vein injection; 2 min later, mouse brains and spinal cords were dissected and immediately embedded in OCT for sectioning. 10 μm‐thick frozen sections were prepared, and fluorescent images captured under an LSM 510 META confocal microscope (Carl Zeiss, Thornwood, NY, USA).

### Histology and immunohistochemistry staining

Immunized EAE control mice (*n* = 5) and CGS‐21680 treated animals (*n* = 5) at 21 dpi were terminally sacrificed. The animals were anaesthetized with isoflurane and transcardially perfused with saline solution. The brains and spinal cords were isolated and fixed in 4% paraformaldehyde. Then, paraffin‐embedded MS brain and EAE mouse (spinal cord & brain) specimens were cut into 5 μm sections for hematoxylin and eosin (H&E), Luxol fast blue (LFB), and immunohistochemical (IHC) staining.

For IHC staining, slides were first permeabilized with 0.2% Triton and blocked using 5% normal goat serum (Invitrogen, Carlsbad, CA, USA). Then, the slides were incubated with primary antibodies (anti‐A2aR, anti‐CD45, and anti‐CD31; Abcam, 1:100) at 4°C overnight, followed by HRP‐conjugated secondary goat‐anti‐mouse or anti‐rabbit antibodies for 1 h at room temperature. Signals were finally revealed by the UltraView DAB kit.

### Cell culture and treatment

Mouse brain endothelial cells (bEnd.3; ATCC) were cultured in high glucose DMEM supplemented with 10% FBS. Cells were synchronized with DMEM containing only 1% FBS for 24 h, and grown to 80% confluency before treatment with Th1 cytokines (Including IL‐1, TNF‐α, and IFN‐γ; 10 ng/ml, respectively) or Th1 cytokines (10 ng/ml, respectively) plus CGS‐21680 (10 nM). CGS‐21680 doses were based on previous in vitro studies 11.

### Immunofluorescence and laser confocal microscopy

bEnd.3 cells were cultured in eight‐well chamber slides. At 80% confluency, the cells were fixed with 3.7% paraformaldehyde. For F‐actin staining, cells were incubated with FITC‐phalloidin (Life Technologies, Carlsbad, CA, USA) for 30 min; for immunocytochemistry, cells were incubated overnight at 4°C with rabbit anti‐Claudin 5 (1:500; Dako), rabbit anti ZO‐1 (1:1000; Chemicon), or the appropriate unconjugated isotype control. Primary antibodies were detected with either isotype‐specific anti‐rabbit or anti‐mouse secondary antibodies (1:1000) conjugated with Alexa 488 or 594 (Invitrogen). Negative control samples were performed without the primary antibody. 4′,6‐Diamidino‐2‐phenylindole (DAPI, Invitrogen) was used for counterstaining. Fluorescent images were captured under an LSM 510 META confocal microscope (Carl Zeiss).

### Immunoblotting

bEnd.3 cells were lysed on ice in RIPA buffer. After protein content determination by the BCA protein assay kit, the samples were heat denatured, resolved by 12% SDS–PAGE, and transferred onto nitrocellulose blotting (NC) membranes. The membranes were probed with rabbit anti‐Claudin‐5, anti‐ZO‐1, anti‐MLCK (1:2500, LSBio), anti‐pMLC (1:1000, Cell Signaling Technology, Danvers, MA, USA) and anti‐MLC (1:1000, Cell Signalling Technology), followed by HRP labeled anti‐rabbit or anti‐mouse secondary antibodies (1:100, Vector). Protein signals were detected by enhanced chemiluminescence (Pierce), with β‐actin used as an internal control.

### Statistical analysis

GraphPad Prism 5 was used to assess significance of all data. One‐way ANOVA was used to assess multiple groups, and unpaired two‐tailed Student's *t*‐test for group pair comparisons. Statistical significance was set at *p* < 0.05.

## Results

### A2A receptor is expressed in the endothelium of MS lesions

**Figure 1:**
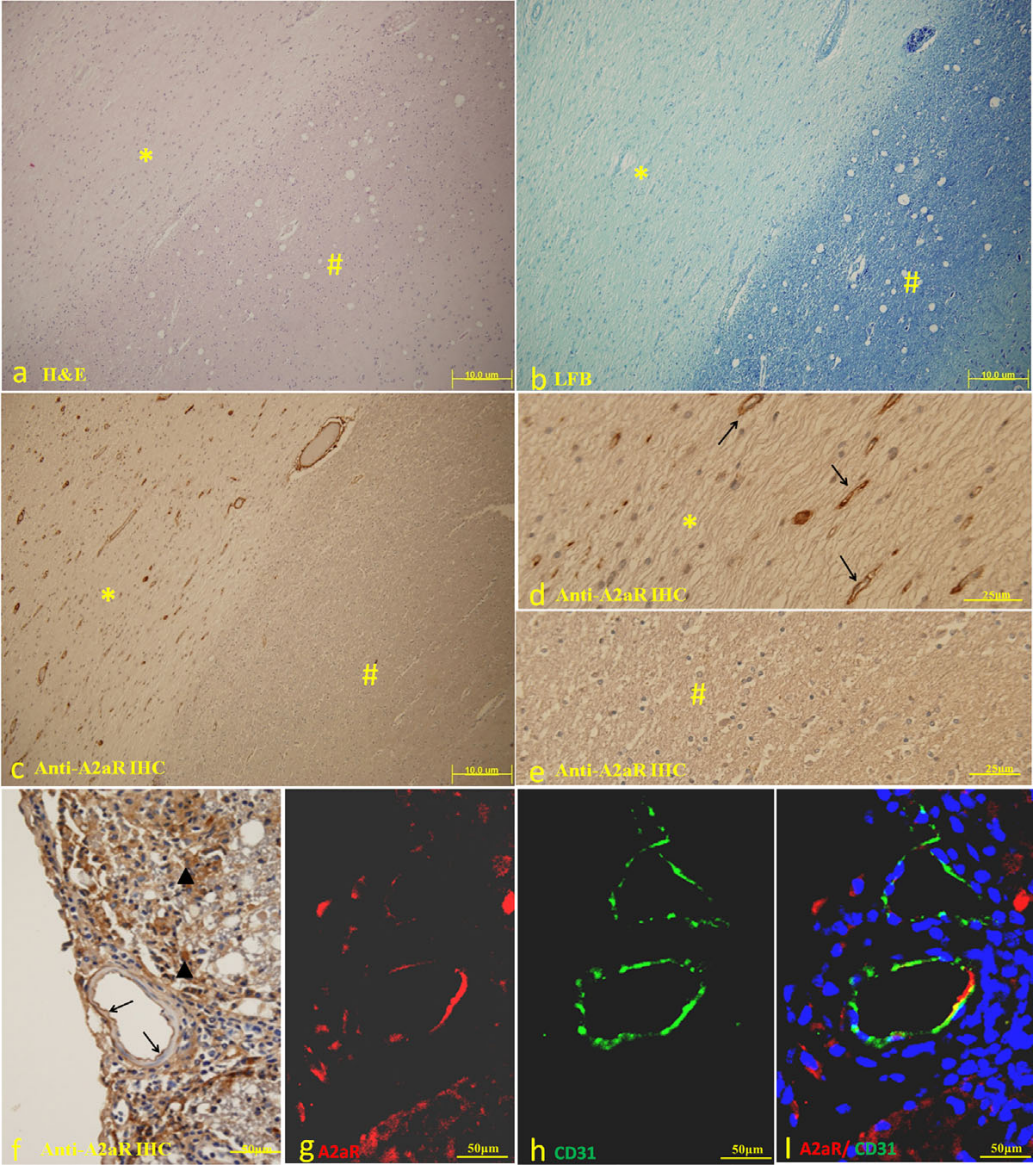
(a) A low power overview showing a partial view of an MS lesion by Hematoxylin‐eosin (HE) staining *demyelination area; ^#^normal appearing white matter (NAWM) (b) the same lesion showed by Luxol fast blue (LFB) staining. *Demyelination area; ^#^normal appearing white matter (NAWM). (c–e) A2A receptor was expressed by the endothelium in de‐myelinated areas, but not in NAWM. *Demyelination area; ^#^NAWM (anti‐A2A receptor immunohistochemistry staining). (f–h) Anti‐A2A receptor immunohistochemistry staining showed that in the EAE lesion, in addition to immune cells, the endothelium also expressed A2A receptors. (i–k) Immunofluorescence staining showed that CD31 positive endothelium were also A2A receptor positive in EAE lesions.

All 28 MS brain specimens showed A2A receptor expression in the brain endothelium of MS white matter lesions, but not in the adjacent normal appearing white matter (Fig. 1a–e). In contrast, A2A receptor was not expressed in the 10 control human brain tissues. Increased A2A receptor staining was also found in inflammatory lesions of EAE mice (Fig. 1f–l). Figure 1 shows the representative expression patterns. These data indicated that A2A receptor was specifically expressed in the endothelium of demyelinated areas.

### A2A receptor activation prevents cytokine‐mediated impairment of tight junction integrity

Tight junction (TJ) proteins are located between brain endothelial cells, assuring BBB integrity. We examined the effects of A2A receptor agonist on expression levels of claudin‐5 and ZO‐1 in bEnd.3 cells after stimulation with Th1 cytokines in the presence or absence of CGS‐21680.

**Figure 2:**
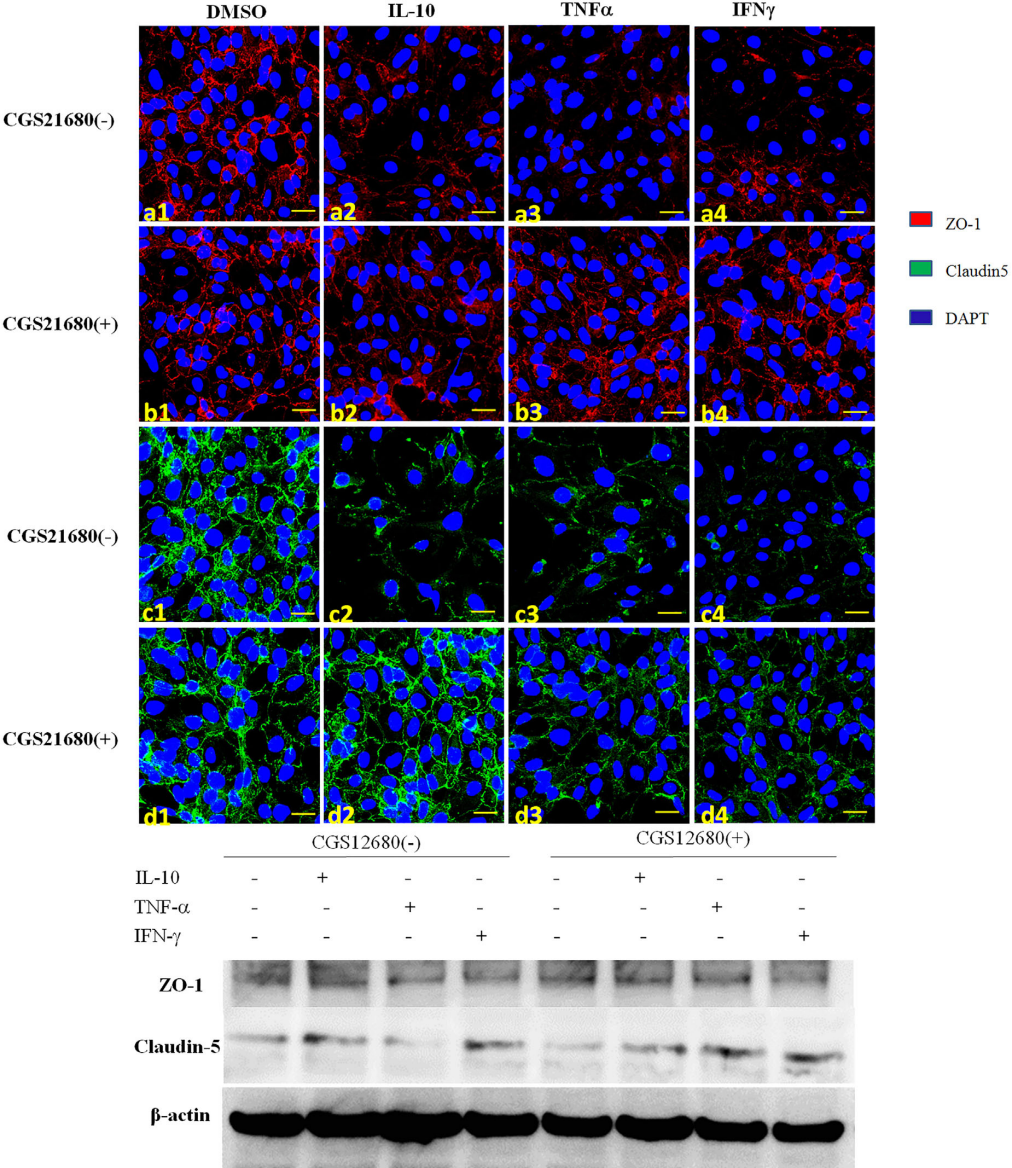
By immunofluorescence, the expression of the tight junction proteins ZO‐1 (a1–4) and claudin 5 (c1–4) was decreased in bEnd.3 cells treated with Th1 cytokines. If the specific A2A receptor agonist CGS21680 was administered, expressions of ZO‐1 (b1–4) and claudin 5 (d1–4) were rescued. Western blot also verified CGS21680 could rescue the expression of ZO‐1 and Claudin‐5 after Th1 cytokine treatment.

Normal bEnd.3 cells exhibited continuous membranous staining of the TJ proteins ZO‐1 and claudin‐5 (Fig. 2a1 and c1). Exposure to Th1 cytokines resulted in disrupted ZO‐1 and claudin‐5 staining at the cell‐cell contact, with a loss of tight junctions after 24 h (Fig. 2a2–4 and c2–4, separately). Treatment with CGS‐21680 partially restored the levels and linear distributions of ZO‐1 and claudin‐5 (Fig. 2b2–4 and d2–4 respectively, with b1 and d1 representing CGS‐21680 control only). Exposure to Th1 cytokines also led to a sustained down‐regulation of ZO‐1 and claudin‐5 at 24 h, which was rescued by CGS‐21680 treatment (Fig. 2e). These results indicated that A2A receptor activation can maintain TJ proteins in Th1 cytokine‐stimulated brain endothelium.

### A2A receptor agonist attenuates Th1‐cytokine induced stress fibers in endothelial cells by reducing MLC phosphorylation

The bEnd.3 brain endothelial cell line has a flat, polygonal morphology at baseline, which is characteristic of an impermeable blood brain barrier 12. Upon activation with Th1 cytokines, cells become elongated with stress fibers and poor barrier function 13. Actin plays a critical role in the characterization and determination of these physiologic states and the transition from one to the other 14. We assessed the role of A2A receptor in endothelial cells under Th1 proinflammatory conditions. Under control conditions, endothelial cells maintained a polygonal shape, and FITC phalloidin staining of F‐actin showed dense peripheral bands (DPBs) around the borders with an interconnected, highly branched web of central actin microfilaments connected to the DBPs (Fig. 2a1). Th1 cytokines, including IL‐1beta, TNF‐alpha, and IFN‐gamma, reduced circumferential actin staining in association with dramatically increased stress fiber formation, particularly in the central regions of cells (Fig. 2a2–4). These changes were partially inhibited by CGS‐21680, an A2A receptor agonist. Figure 2 is representative of three separate experiments.

**Figure 3:**
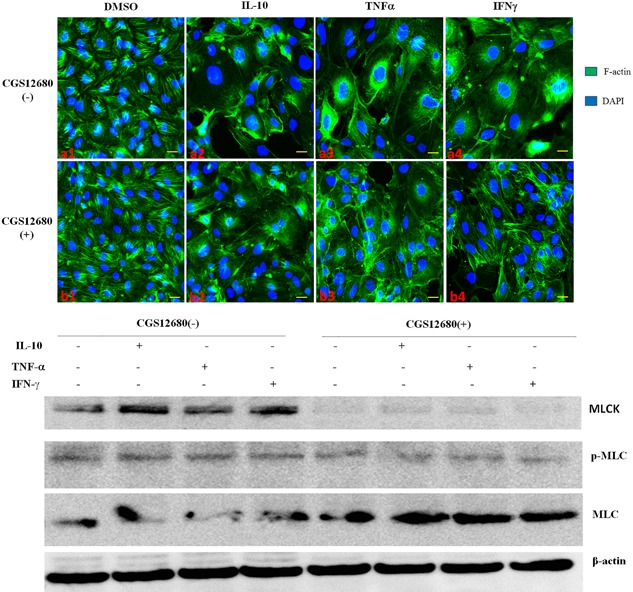
FITC phalloidin staining of F‐actin in bEnd.3 endothelium showed that Th1 cytokines decreased circumferential actin amounts in association with a dramatic increase of stress fiber formation, particularly in the central regions of cells, to various degrees (a1–4). These changes were partially inhibited by CGS21680 (b1–4). MLCK, p‐MLC and total MLC levels were detected by Western blot; CGS21680 reduced MLCK expression, likely further reducing phosphorylated MLC. The cytokines were used at 10 ng/ml and CGS21680 at 10 nM.

We next explored the mechanism by which A2A receptor activation reduces the Th1 cytokine‐mediated inflammatory response in bEnd.3 cells. It is known that Th1 cytokines modulate endothelial cell morphology by activating myosin light chain kinase (MLCK), which phosphorylates MLC (p‐MLC), disrupting the actin‐myosin interaction 15. Therefore, MLCK activation also results in impaired TJ integrity 16. Treatment with Th1 cytokines of bEnd.3 cells resulted in increased p‐MLC and MLCK protein expression levels. Concomitant treatment of bEnd.3 cells with CGS‐21680 reduced the expression of total MLCK and p‐MLC (Fig. 3).

### In EAE, specific A2A receptor agonist decreases BBB permeability, inhibits neuroinflammation, and ameliorates the pathological and clinical disease severity

**Figure 4:**
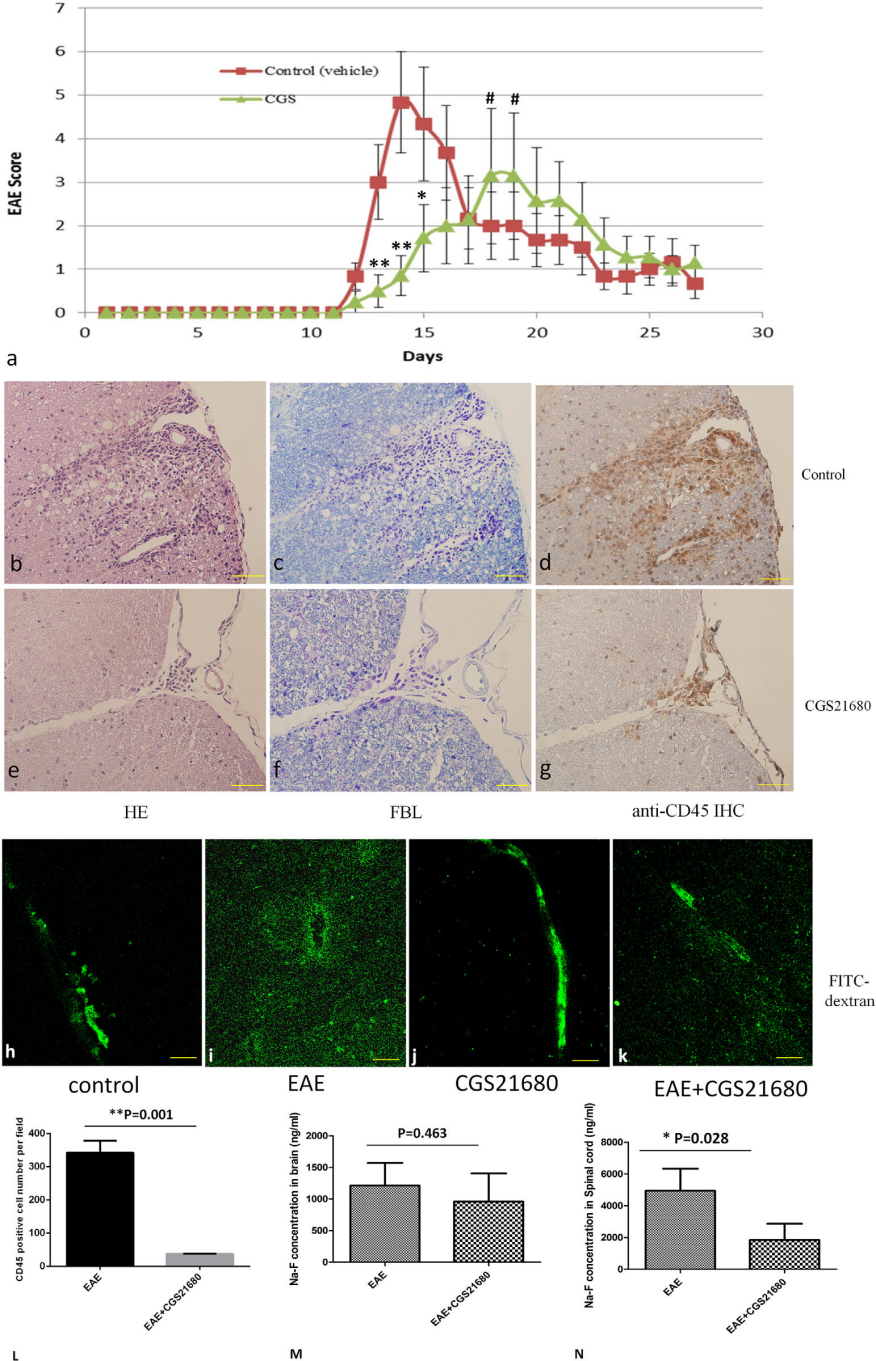
CGS21680 reduces EAE induced motor paralysis. Average EAE scores are displayed relative to the motor deficits observed (a). Significant late onset (*p < 0.05, **p < 0.01) and average maximum EAE score (^#^ *p* < 0.05) reduction in the severity of motor paralysis were observed after CGS21680 therapy. Error bars indicate SEM. Pathologically, the CGS21680 treatment group showed alleviated tissue damage, demyelination, and CD45 positive lymphocyte infiltration (b–g and l). FITC‐dextran as a tracer was used to study BBB permeability. Normally, the fluorescence tracer was limited to the lumen of blood vessels (h); in EAE mice, the tracer leaked into the parenchymal space (i). In CGS treated mice, FITC‐dextran was partially limited to the blood vessel lumen (j and k). Na‐F amounts showed no change in the brain between EAE control and CGS groups (m), but were significantly decreased in spinal cord samples from the CGS treatment group compared with EAE control mice (n).

To assess the role of A2AR signaling during EAE progression, we administered the A2AR agonist CGS‐21680 (50 μg/kg, i.p., daily, from 1 dpi) to EAE mice, which were scored daily. As shown in Figure 4a, A2A receptor agonist significantly decreased paralysis in EAE mice as determined by delayed disease onset and low average maximum EAE score. Reduced disease severity in EAE mice treated with CGS‐21680 also correlated with decreased spinal cord inflammation and demyelination (Fig. 4e and f compared to Fig. 4b and c). To assess CNS lymphocyte infiltration during EAE in mice treated with CGS‐21680, spinal cord sections were examined for the presence of CD45^+^ leukocytes by immunohistochemistry. CGS‐21680 treated EAE mice had significantly less spinal cord CD45^+^ cells compared with control mice (Fig. 4d and g).

We also evaluated the effects of A2AR activation on BBB permeability in vivo. Fluorescein‐labeled dextran (FITC‐dextran) was used as a tracer in morphological studies of vascular permeability. In control mice, the tracer was clearly confined within the lumen of spinal cord microvessels (Fig. 4h). In contrast, EAE affected mice showed FITC‐dextran as diffuse fluorescence along the perivascular space, and as fluorescent granules in neuropils (Fig. 4i). In CGS treated EAE mice, the amounts of tracer that diffused into the brain parenchyma were reduced (Fig. 4k). BBB permeability was quantitated by injection of the small molecular tracer Na‐F (376 Da) into EAE mice; Na‐F fluorescence levels in the brain and spinal cord were measured. Tracer fluorescence in the spinal cord was decreased by about 2.2 times in EAE mice treated with A2A receptor agonist compared with the control EAE group (*p* < 0.05) (Fig. 4m). In contrast, CGS treatment had no effect on BBB permeability (Fig. 4l). Taken together, these findings indicated that A2A receptor agonist provided a protective barrier effect, thereby limiting the degree of permeability induced by inflammation.

## Discussion

Th1 cytokines promote BBB permeability; previous studies suggest that the independent IFN‐γ and TNF‐α signaling pathways converge at MLC phosphorylation to affect BBB permeability. IFN‐γ activates the small GTPase RhoA and increases the expression of Rho associated kinase (ROCK), which in turn phosphorylates and activates myosin light chain (MLC) 17. TNF‐α stimulates NF‐κB to increase myosin light chain kinase (MLCK) transcription, which further correlates with increased MLCK protein levels, MLC hyper‐phosphorylation, and paracellular permeability. Activated MLCK phosphorylates MLC and decreases TJ protein amounts, leading to cytoskeletal rearrangement and impairment of TJ integrity 18. After treatment of bEnd.3 cells with Th1 cytokines, MLC phosphorylation levels were increased, with decreased tight junction protein expression and increased actin contractility.

Adenosine has been reported to enhance endothelial barrier function and attenuate oxidant‐induced barrier dysfunction through A2 receptors 19. However, Do‐Geun Kim 20 and Xihui Gao 21 reported that A2A receptor activation in normal endothelial cells promotes BBB permeability in a transient, rapid, and reversible way. Here, we showed that under pathological conditions, in Th1 cytokine‐treated brain endothelial cells, the A2A receptor specific agonist CGS‐21680 conferred direct BBB protection, by precluding MLC phosphorylation and stabilizing the tight junction proteins ZO‐1 and claudin‐5. Additionally, CGS‐21680 treatment helped maintain endothelial cell shape by preventing Th1 cytokine‐induced formation of stress fibers in the cell. These different effects of A2A receptor activation may be related to its ability to inhibit MLCK mediated MLC phosphorylation. We observed in vivo that the selective A2A receptor agonist maintained barrier integrity as reflected by lower levels of fluorescence‐labeled high‐molecular‐weight dextran (40 kDa) entering the parenchyma of the spinal cord in CGS‐21680‐treated EAE mice than in the untreated EAE group. We used Na‐F in the parenchyma as an indicator of BBB permeability. Interestingly, CGS‐21680 decreased Na‐F amounts in the spinal cord of EAE mice, which correlated with reduced lymphocyte infliction and alleviated disease severity. These data indicate that A2A receptor activation plays a regulatory role in lymphocyte trafficking into the CNS, and protects BBB integrity in EAE.

A2A receptor activation has shown both protective and detrimental effects in different diseases depending on the nature of tissue injury and the associated pathological conditions, as reviewed by Chen JF 22. Recently, Liu reported that A2A receptor significantly suppresses specific lymphocyte proliferation, reduces the infiltration of CD4+ T lymphocytes, and attenuates the expression of inflammatory cytokines, which in turn inhibit EAE progression 23. Furthermore, the A2A receptor‐specific agonist Lexiscan, an FDA approved drug, was shown to be beneficial in the treatment of inflammatory bowel disease, liver ischemia‐reperfusion, and lung injury.

Basal expression of A2A receptors in endothelial cells has also been observed in vitro in various cells, for example, brain microvascular‐, human umbilical vein‐ and pulmonary artery‐ endothelial cells 24. In vivo, A2A receptor is expressed in the spleen and thymus at high levels, and in the heart, lung and blood vessels at low levels in humans 25. Under normal physiological conditions, brain blood vessels express very low A2A receptor levels. Using reverse transcription‐polymerase chain reaction, A2A receptor has been detected in all brain regions 26. Trincavelli reported that IL‐1 and TNF‐α up‐regulate A2A receptors in rat PC12 cells 17; more recently, Nguyen found that Th1 cytokines (IL‐1, TNF‐α, and IFN‐γ) significantly enhance A2A receptor expression in human microvascular endothelial cells 6. We found strong immunopositive A2A receptor signals on the vasculature of MS and EAE lesions. The presence of A2A receptor offers an opportunity for targeting the receptor with a synthetic and selective A2AR agonist, monitoring its effect on neuroinflammation. Ingwersen found that A2A receptor provides anti‐inflammatory effects in T cells, and thus protection at early disease stage 27. A2A receptor activation at the onset of EAE, as demonstrated above, could protect BBB integrity, indicating a regulatory role for A2A receptor in BBB function in demyelinated diseases of the CNS 28, 29.
